# Impact of Environment, Life Expectancy and Real GDP per Capita on Health Expenditures: Evidence from the EU Member States

**DOI:** 10.3390/ijerph182413176

**Published:** 2021-12-14

**Authors:** Yilmaz Bayar, Marius Dan Gavriletea, Mirela Oana Pintea, Ioana Cristina Sechel

**Affiliations:** 1 Department of Public Finance, Faculty of Economics and Administrative Sciences, Bandirma Onyedi Eylul University, Bandırma 10200, Turkey; yilmazbayar@yahoo.com; 2 Department of Business, Business Faculty, Babes-Bolyai University, 400084 Cluj-Napoca, Romania; 3 Finance Department, Faculty of Economics and Business Administration, Babes-Bolyai University, 400084 Cluj-Napoca, Romania; mirela.pintea@econ.ubbcluj.ro; 4 Faculty of Automotive, Mechatronics and Mechanical Engineering—Technical University of Cluj-Napoca, 400641 Cluj-Napoca, Romania; ioana.sechel@auto.utcluj.ro

**Keywords:** health expenditures, environment, life expectancy, real GDP

## Abstract

This research explores the impact of environment, life expectancy, and real GDP per capita on health expenditures in a sample of 27 EU member states over the 2000–2018 period through causality and cointegration analyses. The causality analysis revealed a significant unilateral causality from variables of greenhouse gas emissions, life expectancy, and real GDP per capita to health expenditures. In other words, greenhouse gas emissions, life expectancy, and real GDP per capita had a significant impact on health expenditures in the short run. The cointegration analysis indicated that life expectancy and real GDP per capita had a significant positive impact on health expenditures at the overall panel. On the other side, the country level cointegration coefficients revealed that life expectancy had a considerable positive impact on health expenditures, real GDP per capita had a moderate positive impact on the health expenditures in most of the countries in the panel, but the environment proxied by greenhouse gas emissions had a low positive or negative impact on the health expenditures in a limited number of countries.

## 1. Introduction

Health care expenditures are seen by Grossman, in his health investment theory, as a key investment in both health and productivity, while health is one of the most important factors of human capital and a relevant force that drives economic growth. Health is and needs to be seen as a criterion for economic performance that leads to economic growth [1], seen today in the context of sustainable development. The idea of this paper started from the concept of sustainable development which, according to the definition by the World Commission on Environment and Development (WCDE) [2], is meeting the needs of the present without compromising the needs of the future, being based on three pillars: economic, social, and environmental. The theme chosen in this study addresses several issues of interest in terms of determinants of health care expenditures such as environmental issues measured through greenhouse gas emissions, life expectancy and real GDP per capita that impact health spending at the European level in the current pandemic context. An increase in health care expenditures needs to be seen as a major concern for governments, and understanding the determinants of it can help decision makers to develop appropriate policies.

There are many factors that influence both population health and healthcare expenditures, such as income level, pollution related to the level of industrialization, environmental quality, etc. Providing quality health care services should be one of the most important objectives of governments because they can lead to improved life expectancy, labour productivity, and social and economic welfare. Considering the importance of this sector in each country, in recent years, we found a growing number of studies attempting to investigate the determinants of health care expenditures, such as Jerret et al. [3], Boachie et al. [4], Abdullah et al. [5], and Ullah et al. [6].

The deterioration of environmental quality all over the world has a significant impact on what we call ‘healthy living’. Researchers are recognizing health as a ‘public good’ [5]; thus, as authors emphasize, it is not at the mercy of the ‘invisible hand’.

As a first area of research, we chose to analyse the impact of environmental factors measured through greenhouse gas emissions on healthcare expenditures, knowing that pollution has serious negative consequences on the population’s health, leading to different diseases [7]. Although we found many studies regarding the determinants of healthcare expenditures (HCE) [4,5,8,9,10], the literature concerning the relationship between environmental quality (measured through different indicators) and HCE is still limited, despite the important implications of the increased concentrations of greenhouse gases in the atmosphere on life on our planet. 

As a second area of research, we focused on the relationship between life expectancy and health expenditure. In recent years, life expectancy among different countries has increased due to factors such as development and progress in healthcare services, the usage of advanced technology, and, not at last, improved living standards. Thus, currently, the healthcare needs of the population are increasing, and, in some countries, we witness a faster growth of healthcare expenditure compared to income growth (EU countries, OECD countries, G7 countries) [11]. By addressing the relationship between life expectancy and health expenditure, we analyse the relationship between the inputs and the outputs of healthcare systems. Life expectancy reflects the outputs of the system, while healthcare expenditure reflects the inputs of the system. 

Our third area of research focuses on the relationship between economic growth measured through GDP per capita and health expenditure. We expect, from our study, to obtain a positive correlation between growth in real GDP and healthcare expenditures. It is obvious that a rise in income will determine the population to be able to spend more on health. In this study, we see real GDP as both a measure of economic growth and income. 

Different studies confirm that GDP is one of the main impact factors that influence the variations in health expenditures across countries [12], particularly in developing ones [13]. 

A good knowledge of the relationship between the three factors (environment, life expectancy and income) and the level of health expenditure can be used in making decisions related to the efficient use of financial resources of European states to increase life expectancy and reduce pollution. In this study, we investigated the effect of the three factors on health spending through panel cointegration as well as causality analysis and application of an econometric model using Stata 14.0 and Eviews 10.0. The study targets to contribute to the empirical literature in three ways. First, a limited number of scholars have analysed the effect of environment and life expectancy on health expenditures for the EU countries. Furthermore, the study proxies the environment with greenhouse gas emissions per capita, unlike the common use of CO_2_ emissions in the related empirical literature. Third, the employment of a cointegration test with structural break enables us to consider the recent financial crises in the analyses. 

This research explores the impact of environment, life expectancy, and real GDP per capita on health expenditures in a sample of 27 EU member states over the 2000–2018 period through causality and cointegration analyses. The study is organized as follows: Section 2 presents a brief literature review regarding the chosen topic, Section 3 reflects data and the empirical methodology used, Section 4 explains the main results of the study and finally, Section 5 presents the conclusions of our research.

## 2. Literature Review

The purpose of this study is to empirically examine the effects that environmental quality measured through greenhouse gas emissions, life expectancy and real GDP per capita have on health expenditures. In what follows, we try to present the literature considering the pairwise relationship between the variables considered in our study. 

First, we address the studies concerning the relationship between environmental quality and healthcare expenditure. Most of the literature has revealed a significant positive interaction between CO_2_ emissions and health expenditures, but several scholars such as Boachie et al. [4] and Qureshi et al. [14] have indicated insignificant interactions between the two variables. 

Jerret et al. [3] conducted a cross-sectional data analysis from 49 counties of Ontario, Canada using a sequential two-stage regression model to establish the relationship between environmental quality measured through total pollution output (emissions) and health expenditures and found a significant association between the two variables. Their main result shows that countries with higher pollution emissions have higher per capita health expenditures. In a study published in 2006 by Kiymaz et al. [15], using a panel unit root and cointegration analysis, researchers found that in some provinces of China, environmental factors such as pollution have a positive impact on public health spending. Using a panel cointegration approach, Narayan and Narayan [16] investigated the relationship between environmental quality and per capita health expenditures in eight OECD countries, namely Denmark, Austria, Ireland, Iceland, Spain, Norway, Switzerland, and the United Kingdom and revealed that CO_2_ emissions, per capita income, and per capita health expenditure are cointegrated. 

Abdullah et al. [5] performed a cointegration analysis by using health expenditures and greenhouse gas emission variables and found a long-run relationship between the two elements. Ullah et al. [6] conducted a study using time series data from 1998–2017, and the results indicated that the increase of trade volume leads to an increase in CO_2_ emissions, which in turn are driving up health spending. A similar study was conducted by Odusunya et al. [17] in Nigeria, which revealed a positive impact of CO_2_ emissions on health care expenditures.

To test the causal relationship between CO_2_ emissions, health expenditures, and GDP growth, Chaabouni and Saidi [18] conducted a study for 51 countries (grouped according to income level) using simultaneous equations models and generalized method of moments (GMM). The results revealed a bidirectional causality between CO_2_ emissions and GDP per capita and between health expenditures and economic growth for all three groups of countries, while a unidirectional causality from CO_2_ emissions to health spending was discovered, except for the groups of low-income countries. Moreover, health plays an important role in economic growth, yet it has a limited impact in cases of increasing levels of environmental degradation.

Apergis et al. [9] provided an empirical analysis of the short- and long-run effects of CO_2_ emissions on health care expenditures across 50 the United States for the period between 1966 and 2009, using various statistical models and indicating the fact the increasing CO2 emissions led to increasing health care spending. The results showed that the effect of carbon dioxide emissions on the US health care system was stronger in states where higher amounts were spent on health care. The main message of the study concludes that the tangible health benefits can be associated with US carbon emissions reduction policies. This study is important because the US was the second largest emitter of CO2 in 2019 after China and ahead of the European Union.

In 2017, Zhi-Nan et al. [19] conducted a study on the dynamics of the relationship between environmental pollution, economic development, and public health in 30 Chinese provinces, using data from 2002–2004, and the results revealed a negative effect of environmental pollution on public health. Moreover, they found that economic and social factors have a direct effect on public health and indicated that GDP per capita has substantial negative implications on perinatal mortality, education, and medical conditions. A stable long-term balance between public health, environmental pollution, economic growth, and health services across the country has been identified. 

Chen et al. [10] analysed the relationship between CO_2_ emissions and HCE in 30 provinces of China for the period 2005–2016 using Bayesian quantile regression and argued that CO_2_ emissions act as a driving factor of HCE, while the income variable has a greater influence on HCE.

Although most previous studies have found a relationship between CO_2_ emissions and health expenditures, some researchers found no association between variables [4,14]. The study of Qureshi et al. (2015) [14] was conducted on five Asian countries during the period 2000–2013, revealing no cointegration relationship between CO2 emissions and healthcare expenditures. In their study, Boachie et al. [4] focused on determinants of healthcare expenditures in Ghana and found that CO_2_ emissions (as a measure of pollution) have a positive but insignificant impact on healthcare spending, explaining this result by the low level of Ghana’s industrialization.

Second, we focused on studies that address healthcare expenditure in relation to life expectancy. Bilgel and Tran [20] studied the impact of life expectancy at birth on healthcare expenditure in Canadian provinces for the period 1975–2002 using a one-way fixed-effect dynamic panel model and noted that one year increase in life expectancy determines a 19% decrease of HCE.

Jakovljevic et al. [21] analysed life expectancy and health expenditure evolution in Eastern Europe over the period 1989–2012 using difference-in-difference and data envelopment analysis and pointed out that EU 2004 members were the best performers regarding the impact of balanced longevity increase on health expenditure growth. Their study also revealed a significant positive correlation between life expectancy and health expenditure in all regions.

Linden et al. [22] studied the relationship between life expectancy at birth and health expenditure using an econometric panel time series method. The study was conducted for a panel of 34 OECD countries grouped in three clusters based on the size of public health expenditures as a share of GDP. The findings differ from cluster to cluster, namely in the group of countries with a large share of public health expenditures in GDP in which authors have discovered a positive correlation between life expectancy at birth and health expenditures, while in the group of countries with low share, although there was identified a positive link between the life expectancy at birth and public health expenditures, the relation was not confirmed for private health expenditures.

Gedikli et al. [23] investigated the relationship between life expectancy and health expenditures in countries like Turkey, Azerbaijan, Kazakhstan, Kyrgyzstan, Tajikistan, Turkmenistan, and Uzbekistan using the panel data approach for the period of 2000–2015 and indicated a significant bidirectional long-term relationship between variables.

Third, we examined the studies concerning the relationship between economic growth/income and healthcare expenditure. Most of the literature has revealed a significant interaction between economic growth and health expenditures, but some researchers such as Devlin and Hansen [24] and Zheng et al. [25] did not find any interaction between these two variables. 

The attention on the interaction between economic growth and health expenditures grew since the study of Joseph Newhouse in 1977 [26], who realized a cross-section analysis of 13 developed countries and revealed a strong positive relationship between per capita GDP and per capita health spending, with an elasticity coefficient above one. The results of this study were confirmed by other researchers, such as Hitiris and Posnett [11], who investigated 20 OECD countries over the period 1960–1987.

According to most studies, GDP per capita is the most important ‘factor’ in explaining healthcare expenditures [12]. Gerdtham et al. [12] conducted an analysis on the determinants of healthcare expenditure in OECD countries for a 20-year period, and the results were in accordance with previous studies [27], namely GDP per capita is highly significant for HCE.

In 2010, Cantarero and Lago-Penas [28] investigated the determinants of healthcare expenditures in 17 Spanish regions for the period 1992 to 2003 and indicated that in regions with higher tax autonomy, regional GDP growth had a direct impact on HCE growth and was found for these regions a weak positive relationship between regional healthcare expenditure and regional income, measured through GDP. 

Bilgel and Tran [20] studied the elasticity of healthcare expenditures to GDP in Canada provinces over a period of 28 years, from 1975 to 2002, and found an income elasticity of HCE lower than one, suggesting that the magnitude of GDP increase effect on HCE is low. 

Based on a panel data analysis of 143 OECD countries over a period of 14 years (1995–2008), Ke at al. [13] tried to determine the factors that drive the growth of healthcare expenditures. Their results are similar with ones of Bilgel and Tran (2011) [20], namely that health care expenditures do not grow faster than GDP. Still, the literature regarding the relationship between GDP and HCE on OECD countries revealed, in many cases, an income elasticity above one [27,29,30,31]. 

Using cointegration and causality tests, Elmi and Sadeghi [32] focused their analyses on developing countries during 1990–2009 and discovered a bilateral causality between economic growth and healthcare expenditures.

Different results were reported by Balaji [33] and Ayuba [34], who found a unidirectional causality running from economic growth to health expenditure in 20 OECD countries and Nigeria. Amiri and Ventelou [35] analysed 20 OECD countries during 1970–2009 by applying a Granger causality test and found a bidirectional relationship between health expenditure and economic growth. Similar results were obtained by Kakihara et al. [36] that analysed 14 OECD countries between the years of 1960–2010 or by Chaabouni and Abdnnadher [37] after applying a Granger causality test on data regarding Tunisia during the period 1961–2008. Their results showed a strong bidirectional causality between economic growth, environmental quality, and health spending.

A bidirectional positive relationship between economic growth and healthcare expenditure was found by Murthy and Okunade [38] in America using the Autoregressive Distributed Lag (ARDL) as empirical methodology, Mladenović, et al. [39] in 28 European countries for a period from 1995 until 2015, Piabuo and Tieguhong [40] in African countries using data from 1995–2015 and Erçelik [41], and in Turkey using data from 1980 to 2015, using the same ARDL technique. Using the ARDL method, the study of Zaidi and Saidi [42] conducted in Sub-Saharan African countries over the 1990–2015 period revealed that economic growth has a positive impact on healthcare expenditures.

The purpose of this paper is to answer the following question: Does environment quality, life expectancy, and income measured through real GDP per capita impact healthcare expenditures in EU member states? 

## 3. Data and Method

The main objective of the study is to analyse the effect of environmental degradation and rising life expectancy—two critical issues of the globalized world on health expenditures. The real GDP per capita as an indicator of the economic development level of the countries was included in the model as a control variable.

The health variable was proxied by current health expenditure per capita expressed in international dollars at purchasing power parity. The independent variable of the environment was represented by greenhouse gas emissions per capita, although the environment has been generally proxied by CO_2_ emissions in the related literature [43,44]. The greenhouse gas emissions per capita indicate total national emissions of the ‘Kyoto basket’ of greenhouse gases, including carbon dioxide, methane, nitrous oxide, and fluorinated gases (F-gases). In this context, they are turned into an indicator expressed in units of CO_2_ equivalents through employing each aforementioned gases’ global warming potential [43]. On the other side, life expectancy was proxied by life expectancy index representing the life expectancy at birth, and economic development was proxied by real GDP per capita based on constant 2010 USD. The symbols of the variables and data sources are displayed in Table 1. All the variables were annual; the variables of health expenditure per capita and real GDP per capita were obtained from World Bank database, and the variables of greenhouse gas emissions per capita and life expectancy index were respectively provided by the Eurostat and the United Nations Development Programme (UNDP) databases. All logarithmic forms of the variables were used in our econometric analyses.

The sample of the study includes 27 EU member states (Austria, Belgium, Bulgaria, Croatia, Cyprus, Czech Republic, Denmark, Estonia, Finland, France, Germany, Greece, Hungary, Ireland, Italy, Latvia, Lithuania, Luxembourg, Malta, Netherlands, Poland, Portugal, Romania, Slovakia, Slovenia, Spain and Sweden), and the study period was from 2000 to 2018, because the variable of health expenditure per capita was available for this period. The statistical packages of Stata 14.0 and Eviews 10.0 were used in the econometric analysis of the study. 

The logarithm of the variables (*LNHEALTH, LNGHG, LNLEI, LNGDP*) were used to establish the research model:

(1) $$LNHEALTH_{it}=\alpha_{0}+\beta_{1}LNGHG_{it}+\beta_{2}LNLEI_{it}+\beta_{3}LNGDP_{it}+u_{it}$$

In the econometric part of the study, the presence of cross-sectional dependence and heterogeneity was first checked, and the stationarity analysis was conducted. Then, the cointegration interaction among health expenditures, greenhouse gas emissions, life expectancy, and real GDP per capita was questioned through the Westerlund and Edgerton [49] cointegration test, considering the findings of heterogeneity and cross-sectional dependency test. The Westerlund and Edgerton [49] cointegration test takes notice of cross-sectional dependency, heterogeneity and the structural break, autocorrelation, and heteroscedasticity. The test statistic is figured out through the following two equations:

(2) $$y_{i,t}=\alpha_{i}+\eta_{i}t+\delta_{i}D_{i,t}+x_{i,t}^{'}\beta_{i}+{\left(D_{i,t}x_{i,t}\right)}^{'}\gamma_{i}+z_{i,t}$$

(3) $$x_{i,t}=x_{i,t-1}+w_{i,t}$$

In the above equations, *i = 1,2, …,N* refers to the cross-sections, *t = 1,2, …,T* refers to the time dimension of the panel. On the other side, $D_{it}$ is the dummy variable; $\alpha_{i}$ and $\beta_{i}$ show constant and slope coefficients before the structural break, and $\delta_{i}$ and $\gamma_{i}$ show the change after the structural break. $w_{i,t}$ is the error term (see Westerlund and Edgerton [49] for the detailed information about the test methodology).

The cointegration coefficients are forecasted with AMG (augmented mean group) estimator of Eberhardt and Teal [50], taking notice of heterogeneity and cross-sectional dependence. The AMG estimator takes notice of the common factors and dynamic effects of the series, yields efficient results for the unbalanced panels, and may be employed in case of endogeneity problem [51].

The Dumitrescu and Hurlin [52] test is the improved version of Granger causality test for heterogeneous panels. The test considers heterogeneity and yields robust results in case of cross-sectional dependence [52]. At the test, *X* and *Y* represent two stationary processes for N units during T period. Therefore, the following linear heterogeneous model is considered:

(4) $$Y_{i,t}=\alpha_{i}+\overset{K}{\underset{k=1}{\sum }}\gamma_{i}^{k}Y_{i,t-k}+\overset{K}{\underset{k=1}{\sum }}\beta_{i}^{k}X_{i,t-k}+\varepsilon_{i,t}$$

In Equation (4), *K* is optimal lag length. The null hypothesis of the test is that there is no causality from *X* to *Y* for all cross-sections. The null hypothesis asserts that there is no significant Granger causality among the series, but the alternative hypothesis asserts there is significant causality at least for one cross-section [52] for the detailed information about the test methodology).

## 4. Empirical Analysis

In the applied part of the research, cross-sectional independency and homogeneity tests were first applied to determine the more robust tests of unit root, cointegration and causality. In this context, LM_adj._ test of Pesaran et al. [53], LM CD of Pesaran [54], and LM test of Breusch and Pagan [55] were conducted, and the findings are displayed in Table 2. The null hypothesis of cross-sectional independency declined at 1% significance level, and in turn, the existence of cross-section dependency was reached.

The homogeneity of the cointegrating coefficients was explored through the adjusted delta tilde test of Pesaran and Yamagata [56], and the findings are displayed in Table 3. The null hypothesis of homogeneity declined at 1% significance level, and in turn, cointegrating coefficients were discovered to be heterogeneous.

The existence of unit root at the series was checked by Pesaran [57] CIPS unit root test regarding the presence of cross-sectional dependency among the countries, and the test findings are reported in Table 4. All the series LNHEALTH, LNGHG, LNLEI, and LNGDP were found to include the unit root at the level but became stationary after the first differencing. In other words, all the series were I (1).

The cointegration relationship among health expenditures, environment, life expectancy, and real GDP per capita was examined through Westerlund and Edgerton [49] cointegration test due to the existence of crises in the study duration, heterogeneity, and cross-sectional dependency, and the findings are displayed in Table 5. The test results for the model with both no breaks and breaks indicated a significant cointegration among the variables because the null hypothesis of no significant cointegration interaction declined at a 5% significance level. Furthermore, the structural breaks revealed the significant impact of both global financial crises and Eurozone sovereign debt crises, as seen in Table 5. 

The cointegration coefficients were estimated by augmented mean group (AMG) estimator of Eberhardt and Bond [51] due to the presence of cross-sectional dependency and heterogeneity, and the findings are displayed in Table 6. The panel cointegration coefficients indicated that life expectancy and real GDP per capita had a significant positive impact on health expenditures.

Furthermore, the individual cointegration coefficients revealed that greenhouse gas emissions had a positive weak effect on health expenditures in Ireland, Portugal and Spain and a negative weak effect on health expenditures in Austria, France, Italy, and Poland. On the other side, life expectancy had a considerable positive impact on the health expenditures in Austria, Belgium, Bulgaria, Estonia, Finland, Germany, Ireland, Latvia, Lithuania, Slovenia, and Sweden. Lastly, real GDP per capita had a moderate positive impact on the health expenditures in Cyprus, Denmark, Estonia, Finland, France, Germany, Greece, Italy, Latvia, Luxembourg, Poland, Portugal, Romania, Slovak Republic, and Slovenia.

The premise from which we started our research is confirmed, namely the environmental quality, life expectancy, and GDP per capita impact health expenditure, as we explained before. Our study confirms the results from the revised literature; namely, one of the most important determinants of HCE expenditure is GDP per capita [11,12,26,27,33,34,35,36,37,38,41,42]. 

Regarding the impact of GHG emissions on HCE, our results divide the countries into two groups: countries with a positive impact of CO_2_ on HCE (Ireland, Portugal and Spain) and countries with a positive weak impact of CO_2_ on HCE (the rest of the countries considered in the study). This result also confirms the findings of other researchers that differences among the countries are given by the degree of industrialization. For example, Boachie et al. [4] explained their findings on Ghana (positive but insignificant impact of CO_2_ emissions) by the low level of Ghana’s industrialization. Differences among countries are given by the level of HCE and its percentage in GDP but also by the dynamics of GDP. In order to fully understand and explain the results obtained, in our next research, we will group the countries considering the level of income and the share of HCE in GDP. Regarding the relationship between life expectancy and healthcare expenditures, most of the literature found a connection between these two variables [21,22,23].

The causality among health expenditures, greenhouse gas emissions, life expectancy, and real GDP per capita was analysed through Dumitrescu and Hurlin [52] causality test, and the findings are displayed in Table 7. The causality analysis revealed a significant unilateral causality from the variables of greenhouse gas emissions, life expectancy, and real GDP per capita to the health expenditures. In other words, greenhouse gas emissions, life expectancy, and real GDP per capita had a significant impact on health expenditures.

A unidirectional causal relationship between CO_2_ emissions and healthcare expenditures was validated through various studies such as Chaabouni and Saidi [18], Erdogan et al. (China) [58] since other studies identified a bidirectional causality [8,18] or no significant causality [58].

According to the results obtained by Piabuo and Tieguhong [40], there is a unilateral causality running from health expenditure to life expectancy since our findings suggested a causality running in the opposite direction.

Our results also identified a one-way positive causality relationship from economic growth to healthcare expenditures that confirm Balaji [33] and Ayuba [34] conclusions. Other studies conducted by Amiri and Ventelou [35] Kakihara et al. [36], Chaabouni and Abdnnadher [38] revealed a bidirectional causality between variables.

## 5. Discussion

The main purpose of this research was to investigate the impact of environmental degradation and rising life expectancy on health expenditures. The empirical analysis is based on a sample of 27 EU member states over the period 2000–2018. We found that all variables used in the econometric analysis are integrated of order one; therefore, we can confirm the existence of a long-run relationship between health expenditures, environmental pollution and life expectancy.

Analysing the individual cointegration coefficients, we found mixed results: greenhouse gas emissions had a positive weak effect on the health expenditures in countries such as Ireland, Portugal, and Spain, this result being supported by the study of Odusunya et al. [17], Ullah et al. [6] and a negative weak effect in Austria, France, Italy, and Poland.

In cases where environmental pollution is rising, our results indicate that, for some countries, the results are different. The divergence in results can be explained by the fact that different studies used different variables to proxy environmental pollution. Most of the previous researchers included in their studies only the CO_2_ emissions; our analyses used the greenhouse gas emissions per capita.

Moreover, our results indicate that life expectancy had a considerable positive impact on health expenditures in most of the countries, which can be explained by the fact that we conducted a study that included developed countries characterized by high living standards that increase the longevity of people and reduce mortality risks. Lifestyle changes and technological advances in the medical sector extend human lifespan but in turn increase health-related costs.

Our results also indicate that real GDP per capita had a moderate positive impact on health expenditures in most countries included in the sample, and these results can be explained by the fact that by having higher income per capita, countries can increase public health expenditures.

The causality analysis revealed a unidirectional causality running from real GDP per capita, life expectancy, and GHG emissions to the health expenditures. An increase in GDP per capita will raise health expenditures, which suggests that economic development, beside its positive effects, will influence negative health factors that in turn will generate growth in health spending. This is an important message for policy makers to make efforts to find proper solutions to limit GHG emissions since environmental pollution will raise health expenditures.

Over the past decades, life expectancy has increased across EU countries, and concomitant health spending, based on the fact that for older people, higher costs of medical treatments or care services are necessary.

## 6. Conclusions

Considering the importance of health in the development of human capital, the identification of the main healthcare expenditure determinants has important implications for both researchers and policy makers. Many studies have examined the link between different socio-economic indicators such as income, globalization, inflation, life expectancy, level of industrialization, and healthcare expenditures, but in the context of sustainable development, that requires the usage of present resources, and, taking into consideration the needs of the future generation, environmental dimensions need to also be addressed. Thus, our study explores the effect of environment quality measured through greenhouse gas emissions per capita, life expectancy and real GDP per capita on health expenditures in 27 EU member states during the 2000–2018 period. The health status of a population has a great impact on work productivity and efficiency, capacity to learn and the ability to grow on various levels. As many researchers have concluded, the pillar of economic growth in any state is the health sector.

Our results are useful for policy making regarding both public health expenditure and investments in European countries, especially in the present context of the world pandemic. Our findings revealed that greenhouse gas emissions, life expectancy, and real GDP per capita had a significant impact on health expenditures in EU countries. Considering this, policy makers should be aware that any policies for improving life expectancy through the overall healthiness of the population would lead to an increase in healthcare expenditure, while any policies aimed to increase real GDP can positively impact investments in the healthcare sector. We chose to approach greenhouse gases because it has a severe impact on climate change and on human health. The increase in environmental degradation is due to unsustainable economic growth; thus, any government should prioritize sustainable economic growth. Policy makers should consider the importance of health care expenditures as an essential driver of economic growth because of their impact on human capital development.

In conclusion, our study reveals strong links between environmental quality, income, life expectancy, and healthcare expenditures, as we consider these three elements as key factors in determining healthcare expenditures in European countries. Protecting human health is a necessity, and the global health crisis generated by the spread of COVID-19 has demonstrated that no country is fully prepared to adequately handle a pandemic. Investigation of factors that can improve the health sector is a necessity and can help state and local public health authorities to find solutions to make this sector more resilient. There is a real challenge for any country to achieve sustainable economic growth that in turn will stimulate governments to increase spending on health.

In terms of the limitations of our research, we need to emphasize that our analysis focused on data collected from 27 EU member states; therefore, future studies should extend the size of the sample. In addition, since our work does not include any developing country, future research should consider countries at different levels of development.

## Figures and Tables

**Table 1:** Data Description.

Variables	Description	Source
HEALTH	Current health expenditure per capita (PPP current international USD)	[45]
GHG	Greenhouse gas emissions per capita (tonnes of CO2 equivalent per capita)	[46]
LEI	Life expectancy index	[47]
GDP	Real GDP per capita (constant 2010 USD)	[48]

**Table 2:** Results of Cross-sectional dependency tests.

Test	Statistic	*p* Value
LM	985.4	0.0000
LM adj *	44.57	0.0000
LM CD *	15.83	0.0000

Notes: * two-sided test.

**Table 3:** Results of Homogeneity Tests.

Test	Statistic	*p* Value
$\tilde{\Delta }$	15.944	0.000
${\tilde{\Delta }}_{adj.}$	18.574	0.000

**Table 4:** CIPS panel unit root test results.

Variables	Constant	Constant + Trend
LNHEALTH	−0.812	−2.190
D(LNHEALTH)	−5.657 ***	−3.260 ***
LNGHG	0.383	−1.926
D(LNGHG)	−8.906 ***	−6.087 ***
LNLEI	3.313	6.212
D(LNLEI)	0.19 **	−5.329 ***
LNGDP	−1.049	0.562
D(LNGDP)	−2.589 ***	−1.846 **

Notes: *** and ** are respectively significant at 1% and 5% significance level.

**Table 5:** Results of Westerlund and Edgerton Cointegration Test [49].

Model	$Z_{\varphi }\left(N\right)$	*p* Value	$Z_{\tau }\left(N\right)$	*p* Value
No shift	−3.744	0.000	−6.039	0.000
Level shift	−0.079	0.068	−0.427	0.035
Regime shift	−2.518	0.006	−4.277	0.000
Country	Structural breaks (level shift)		Structural breaks (regime shift)	
Austria	2011		2011	
Belgium	2002		2013	
Bulgaria	2012		2012	
Croatia	2012		2012	
Cyprus	2007		2007	
Czech Republic	2012		2009	
Denmark	2008		2008	
Estonia	2009		2009	
Finland	2013		2013	
France	2002		2002	
Germany	2003		2008	
Greece	2010		2010	
Hungary	2002		2002	
Ireland	2013		2012	
Italy	2005		2005	
Latvia	2008		2015	
Lithuania	2003		2003	
Luxembourg	2011		2011	
Malta	2013		2013	
Netherlands	2013		2013	
Poland	2007		2007	
Portugal	2010		2010	
Romania	2010		2010	
Slovak Republic	2013		2013	
Slovenia	2007		2007	
Spain	2005		2005	
Sweden	2010		2010	

**Table 6:** Long-run cointegrating coefficients.

Country	LNGHG	LNLEI	LNGDP
Austria	−0.530 ***	16.952 ***	0.0201422
Belgium	0.248	12.176 ***	−0.5345347
Bulgaria	0.350	17.415 ***	−0.099
Croatia	0.769	4.613	0.088
Cyprus	−0.085	1.464	0.666 *
Czech Republic	−0.812	4.094	0.535
Denmark	−0.148	2.607	0.569 ***
Estonia	−0.122	4.228 ***	0.546 ***
Finland	−0.096	2.986 *	0.359 **
France	−0.745 ***	2.967	0.733 **
Germany	−0.687 *	8.449 **	1.190**
Greece	−0.181	−7.865	0.995 ***
Hungary	0.230	1.322	0.544
Ireland	0.490 **	8.507 **	−0.303
Italy	−0.387 ***	−0.253	1.167 ***
Latvia	0.259	3.858 ***	0.821 ***
Lithuania	0.081	3.799 **	0.384
Luxembourg	0.224 *	−5.903	0.883 ***
Malta	−0.010	8.466	0.855
Netherlands	−0.246	2.974	0.102
Poland	−0.857 ***	2.632	0.917 ***
Portugal	0.269 *	0.531	1.126 ***
Romania	0.044	−1.332	1.138 ***
Slovak Republic	−0.967	−12.970	1.645 ***
Slovenia	−0.074	3.779 **	0.422 ***
Spain	0.378 *	2.434	0.411
Sweden	0.171	32.155 **	−0.595
Panel	−0.090	4.447 ***	0.540 ***

Notes: ***, **, and * are respectively significant at 1%, 5%, and 10%.

**Table 7:** Results of causality test.

Null Hypothesis	W-Stat.	Zbar-Stat.	Prob.
DLNGHG ↛ DLNHEALTH	4.56542	3.59658	0.0003
DLNHEALTH ↛ DLNGHG	2.06146	−0.64943	0.5161
DLNLIE ↛DLNHEALTH	4.39268	3.30366	0.0010
DLNHEALTH ↛ DLNLIE	2.46440	0.03384	0.9730
DLNGDP ↛ DLNHEALTH	6.16775	6.31368	0.0000
DLNHEALTH ↛ DLNGDP	1.76364	−1.15446	0.2483

## Data Availability

Data sharing not applicable.
